# Between insects and birds: molecular evidence of *Bartonella henselae* DNA in *Rhodnius prolixus* (Stål, 1859) from an insectary and *Cairina moschata* (Linnaeus, 1758) ducks used as triatomine blood meal source

**DOI:** 10.1590/S1678-9946202668036

**Published:** 2026-06-12

**Authors:** Luciene Silva dos Santos, Paulo Eduardo Neves Ferreira Velho, Jader de Oliveira, João Aristeu da Rosa, Osvaldo Campos dos Santos Nonato, Francine Ramos Scheffer, Marina Rovani Drummond

**Affiliations:** 1 Universidade Estadual de Campinas, Faculdade de Ciências Médicas, Laboratório de Pesquisa Aplicada em Dermatologia e Infecção por Bartonela, Campinas, São Paulo, Brazil; 2 Universidade Estadual de Campinas, Faculdade de Ciências Médicas, Departamento de Clínica Médica, Divisão de Dermatologia, Campinas, São Paulo, Brazil; 3 Universidade Estadual Paulista, Faculdade de Ciências Farmacêuticas, Araraquara, São Paulo, Brazil

**Keywords:** Bartonella, Triatominae, Zoonoses, Arthropod vectors

## Abstract

*Bartonella henselae* is the primary human pathogen species of this Gram-negative bacterial genus, associated with several clinical manifestations including cat scratch disease, bacillary angiomatosis, endocarditis, and myocarditis. This pathogen was recently detected in *Triatoma sordida,* a vector of Chagas disease in Brazil. Here, we investigated the presence of *B. henselae* DNA in *Rhodnius prolixus*, another triatomine vector of *Trypanosoma cruzi* maintained in an insectary and in ducks (*Cairina moschata*) used as a blood source for their colony. A total of 84 triatomines and blood samples from 10 ducks were analyzed by nested PCR targeting the *ftsZ* gene of *B. henselae*. Bacterial DNA was detected in four (4.76%) triatomines and in nine (90%) ducks. Sequencing of amplicons from three triatomines and two ducks revealed 100% identity, full query coverage, and highly significant E-values, indicating complete sequence identity to *Bartonella henselae* strain Houston-1. To the best of the authors’ knowledge, this study provides novel evidence of *B. henselae* DNA in *R. prolixus* and *C. moschata*. However, the vector competence of triatomines for transmitting this bacterium to humans and/or ducks remains unclear.

## INTRODUCTION

Bartonellosis is a neglected and potentially fatal disease caused by fastidious, Gram-negative bacteria belonging to the genus *Bartonella*. Among them, *Bartonella henselae* is the primary species associated with human disease, causing clinical manifestations such as cat scratch disease, bacillary angiomatosis, endocarditis, and myocarditis^1^. These bacteria have a high capacity to infect mammals and other animals, and their transmission is commonly associated with blood-feeding arthropod vectors, including fleas, lice, mosquitoes, and ticks^2-4^.

One study revealed that patients with Chagas cardiomyopathy are 40 times more likely to be infected with *Bartonella* spp. compared to healthy controls^5^. Considering the ability of these bacteria to infect a wide variety of mammals, including those serving as reservoirs for *Trypanosoma cruzi*, there is growing concern about the potential sharing of vectors and ecological interactions between these pathogens. This phenomenon has already been reported in other studies^6^.

Evidence of *Bartonella* spp. infection in triatomines has been reported in different geographic regions. Researchers in southern China detected *Bartonella* DNA in 8/22 (36.4%) specimens of *Triatoma rubrofasciata* analyzed^7^. In French Guiana, a novel species, *Candidatus* Bartonella rondoniensis, was described in wild *Eratyrus mucronatus* (13/23, 56.5%)^8^. Recently, *B. henselae* DNA was detected in *Triatoma sordida* collected in peridomiciliary environments in Brazil^9^.

During experimental studies designed to evaluate the vectorial potential of triatomines for *B. henselae*, bacterial DNA was unexpectedly detected in insects from the control group. This finding prompted further investigation into possible infection sources, including triatomines maintained in an insectary and in ducks (*Cairina moschata*) used as their blood meal source, especially because these birds are considered refractory to *T. cruzi* infection^10^.

### Ethics

The research was conducted in compliance with current legislation and was exempt from approval by the Ethics Committee on Animal Use (CEUA / UNICAMP), in accordance with Brazilian federal law Nº 11,794/2008, as it involved invertebrates and donated blood collected from ducks under veterinary supervision at a Brazilian research institute.

## MATERIALS AND METHODS

The ducks were maintained under veterinary supervision at the Central Animal Facility, in accordance with institutional sanitary and welfare guidelines. Initially, the animals were housed in cemented enclosures with access to a water tank. However, following current animal welfare practices and aiming to minimize stress, they were subsequently maintained in areas with access to natural soil and vegetation (earth and grass). This modification favored the birds’ natural behavior and improved their comfort and health indicators.

Blood samples were collected from ten randomly selected *Cairina moschata* ducks via the brachial vein using sterile syringes, in volumes not exceeding 1% of the total blood volume per animal. Immediately after collection, the samples were placed in EDTA-containing tubes and kept refrigerated to preserve cellular integrity. The tubes were then transported to Campinas city, where they were frozen at −20 °C to promote hemolysis and stored until DNA extraction.

Duck blood samples were subjected to both liquid and solid cultures for the isolation of *Bartonella* spp. Liquid enrichment culture was employed to increase bacterial concentration prior to molecular detection. The liquid enrichment culture was prepared based on a modified formulation originally described by Maggi *et al*.^11^ and further detailed by Drummond *et al*.^12^ The enrichment medium consisted of 900 mL of IPL-41 insect medium supplemented with 0.1 mg of NAD (nicotinamide adenine dinucleotide), 1.25 mg of NADP (nicotinamide adenine dinucleotide phosphate), 2 mg of ATP (adenosine triphosphate), 2 mg of pyruvate, and 2 g of yeast extract. For each culture flask, 500 µL of duck blood was added to 2 mL of the enrichment medium. Cultures were incubated under constant agitation at 35 °C in a 5% CO^2^ atmosphere for seven days. After incubation, 1 mL of the culture was used for DNA extraction, and the remaining 1 mL was inoculated onto a solid medium.

The solid medium was prepared by dissolving 6 g of Bordet-Gengou agar base in 117 mL of distilled water and 1.167 mL of glycerol. After autoclaving and cooling to 50 °C, 50 mL of defibrinated sheep blood was added. Prior to use, all batches of defibrinated sheep blood were screened by nested PCR targeting the *ftsZ* gene and by both liquid and solid culture assays, following the same protocol applied to the experimental samples. The *ftsZ* gene has been widely used as a molecular marker for the detection of *B. henselae* in clinical and experimental studies^13^. No *Bartonella* DNA or bacterial growth was detected in any blood batch prior to use.

The medium was dispensed into cell culture flasks with filter caps^14^. Cultures were maintained at 35 °C in a 5% CO^2^ atmosphere with saturated humidity for up to 42 days. Bacterial growth was examined weekly. When colonies showing *Bartonella*-like morphology (small, delicate, Gram-negative coccobacilli) were observed, they were collected for DNA extraction.

Samples were analyzed from 84 *R. prolixus* triatomines originating from colonies maintained at the Triatominae Insectary of the School of Pharmaceutical Sciences, Sao Paulo State University (UNESP), Araraquara city campus, and from donated blood of 10 *C. moschata* ducks used to feed these triatomines.

The triatomines analyzed in this study were routinely fed with defibrinated sheep blood prior to the experiments. All blood batches were previously tested by PCR targeting the *ftsZ* gene and by both liquid and solid culture assays, and confirmed to be free of *B. henselae* contamination^13^. The artificial feeder used was adapted from the model described by Bonnet *et al*.^14^, employing non-lubricated male condoms as the feeding membrane.

Surface decontamination of the triatomines was performed through two rounds of immersion with agitation in 70% ethanol, each lasting 10 min, with drying and replacement of the ethanol between immersions. Following this, the insects were immersed in sterile phosphate-buffered saline (PBS) for 5 min. After drying under a laminar flow hood, the insects were processed individually. Legs, wings (for adults), and antennae were removed from each specimen. The remaining body was bisected along the sagittal plane, with each half placed in separate sterile 1.5 mL tubes. Small insects (non-adults) were placed whole into the tubes. The tubes were then immersed in liquid nitrogen. Using sterile and individual pestles, the insects were ground to the maximum possible fragmentation. Samples were stored at −20 °C for subsequent DNA extraction.

DNA extraction from the insects was performed using the QIAamp DNA Mini Kit (Qiagen^®^), following the tissue protocol. DNA extraction from duck blood was conducted using a sodium chloride-based method with 150 µL of blood per sample, following a previously published protocol^15^. Insect samples were tested by conventional PCR for an endogenous gene from the Reduviidae family targeting the cytochrome *c* oxidase subunit I (COI) fragment, and by nested PCR specific for the *ftsZ* gene of *B. henselae*, as previously described^16,17^. The first amplification generated an expected amplicon of 354 bp, followed by a second amplification producing a 218 bp fragment.

Nested PCR targeting the *ftsZ* gene of *B. henselae* was performed in a final reaction volume of 25 µL. First-round PCR. The first-round amplification used primers BHF (5’- GCCGCAAAGTTCTTTTCATG-3’) and BHR (5’-AGGTGAACGCGCTTGTATTTG-3’). The reaction mixture consisted of 2.5 µL of template DNA, 5 µL of 5× colorless buffer, 2 µL of 25 mM MgCl^2^, 0.5 µL of dNTPs, 1 µL of each 10 µM primer, and 0.125 µL of Taq DNA polymerase (5 U/µL), completed with nuclease-free water to 25 µL. Cycling conditions were 95 °C for 5 min; 40 cycles of 95 °C for 1 min, 56 °C for 1 min, and 72 °C for 1 min; followed by a final extension at 72 °C for 5 min, and a hold at 4 °C. Second-round PCR. The second-round reaction used primers BHS (5’-CAAAACGGTTGGAGAGCGT-3’) and BHA (5’-CGCCTGTCATCTCATCAAGA-3’) and 0.2 µL of the first-round PCR product as a template, in a final volume of 25 µL under the same reaction mixture conditions. Cycling conditions were 95 °C for 5 min; 40 cycles of 95 °C for 1 min, 61 °C for 1 min, and 72 °C for 1 min; followed by a final extension at 72 °C for 5 min, and a hold at 4 °C.

DNA extracted from duck blood and from liquid cultures was tested by PCR targeting the eukaryotic endogenous gene (18S)^18^ and by the same nested PCR protocol specific for *B. henselae*.

Each PCR reaction included a positive control consisting of *B. henselae* DNA sourced from the culture collection of the Adolfo Lutz Institute, and a negative control containing nuclease-free water. Amplicons presenting single bands of the expected size were submitted to commercial Sanger sequencing. Sequence quality was assessed by inspection of electropherograms, considering peak sharpness, minimal background noise, and absence of overlapping signals, in order to confirm amplification specificity. High-quality sequences were subjected to BLASTn analysis against the GenBank database to determine nucleotide identity and confirm species-level identification.

## RESULTS

No amplification was observed in any of the negative controls, while all positive controls showed successful amplification, confirming the reliability of the molecular procedures. All samples from both insects and ducks showed amplification of endogenous genes, demonstrating intact DNA free from inhibitors.

*Bartonella* sp. DNA was detected in 4 out of 84 (4.76%) triatomines and in 9 out of 10 (90%) duck blood samples by nested PCR targeting the *ftsZ* gene. Sequencing of amplicons from three triatomines and two ducks revealed 100% identity, full query coverage, and highly significant E-values in BLAST analysis, showing correspondence with *Bartonella henselae* strain Houston-1 sequences available in GenBank (accession No. CP020742.1).

No *Bartonella* spp. DNA was detected in any liquid enrichment culture samples. In solid culture, a single colony displaying morphology suggestive of the genus *Bartonella*—small, whitish, and delicate, as commonly described in classical culture studies—was selected for molecular confirmation^19^. Microscopic examination revealed Gram-negative coccobacilli compatible with the genus^4^. However, due to insufficient amplicon quality, sequencing could not be performed.

**Figure 1:**
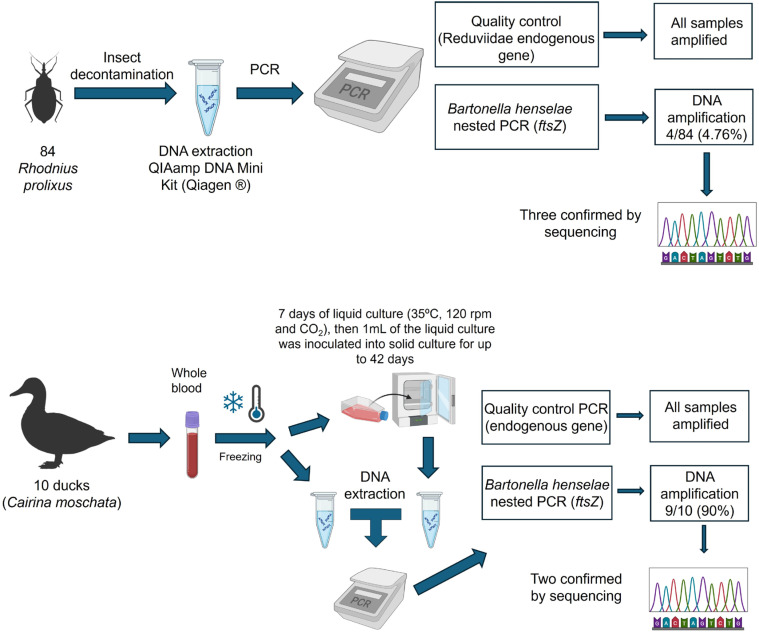
Experimental workflow involving *Rhodnius prolixus* from the Triatominae Insectary of the School of Pharmaceutical Sciences, Sao Paulo State University (UNESP), Araraquara campus, and ducks (*Cairina moschata*) used at the same institution as a blood meal source for these vectors.

Figure 1 illustrates the workflow of sample processing and analysis. Table 1 summarizes the BLASTn metrics of all sequenced amplicons obtained from triatomines and ducks.

**Table 1:** BLASTn metrics of *ftsZ* gene sequences obtained from triatomines and ducks

Host species	Sample ID	PCR target	Closest BLASTn match	GenBank accession Nº	Identity (%)	Query cover (%)	E-value
*C. moschata*	Duck 4	*ftsZ*	B. henselae Houston-1	CP020742.1	100	100	**3e-65**
*C. moschata*	Duck 9	*ftsZ*	B. henselae Houston-1	CP020742.1	100	100	**1e-55**
*R. prolixus*	GC 4E	*ftsZ*	B. henselae Houston-1	CP020742.1	100	100	**2e-23**
*R. prolixus*	GC QD	*ftsZ*	B. henselae Houston-1	CP020742.1	100	100	**3e-52**
*R. prolixus*	GC A1E	*ftsZ*	B. henselae Houston-1	CP020742.1	100	100	**6e-41**

## DISCUSSION

To the best of the authors’ knowledge, this study provides the first molecular evidence of *B. henselae* DNA detection in *R. prolixus*. Previous investigations assessing *Bartonella* spp. in wild *R. prolixus* reported no positive samples, possibly due to the limited number of insects analyzed (n=10)^8^.

No *Bartonella* spp. DNA was detected in the liquid enrichment cultures. This result may be explained by dilution effects combined with the fastidious growth requirements characteristic of these bacteria, as previously discussed in the literature^20^. Additionally, blood samples were stored at −20 °C prior to culture. Although this temperature is suitable for nucleic acid preservation, ultra-low temperatures (≤−70 °C) are generally preferred when long-term maintenance of viable bacteria is required^21^. This factor may therefore have contributed to the absence of detectable bacterial growth.

Birds have already been described as potential reservoirs for *Bartonella* spp.^22,23^; however, this study is the first to associate *Cairina moschata* ducks with the detection of *B. henselae* DNA and to suggest their possible involvement as a blood source for infected triatomines. The ducks used to feed the insects were maintained near the insectary, where they are exposed to vectors and direct or indirect contact with domestic animals (cats [*Felis catus*] are frequently observed in the same area) and wildlife, as the region is relatively wooded. It is known that both *Ctenocephalides felis* fleas and ticks of the genus *Amblyomma*, commonly found throughout Sao Paulo State, can occasionally infest birds^24,25^.

The detection of *B. henselae* DNA in 4 out of 84 triatomines (4.76%) and in 9 out of 10 duck blood samples (90%) suggests the possibility of an environmental transmission interface involving multiple hosts and hematophagous arthropods. However, the presence of bacterial DNA alone does not confirm active infection, vector competence, or reservoir status. The coexistence of positive insects and birds in the same environment primarily raises ecological and epidemiological questions rather than establishing transmission dynamics.

The nested PCR approach employed increases analytical sensitivity and reduces nonspecific amplification. Nevertheless, species-level identification based on a relatively short amplified fragment should be interpreted cautiously, even when sequencing demonstrates complete identity with reference sequences. Sequencing was successfully obtained for three amplicons, and BLAST analysis revealed 100% identity and full query coverage with *B. henselae* sequences, including strain Houston-1, indicating strong similarity with known sequences of this species.

Despite these supportive molecular findings, the reliance on a single genetic locus represents an important methodological limitation. Additional molecular targets or phylogenetic analyses would provide stronger taxonomic resolution and greater confidence in species assignment.

## CONCLUSION

Taken together, these findings represent the first detection of *B. henselae* DNA in both *R. prolixus* and *C. moschata*, highlighting a potential ecological interface between triatomines and avian hosts. However, the detection of bacterial DNA alone does not demonstrate that these organisms function as competent vectors or reservoirs. Further studies are necessary to evaluate the capacity of triatomines to acquire, maintain, and transmit *B. henselae*, thereby clarifying their possible role in the epidemiological cycle of this bacterium.

## Data Availability

The anonymized dataset generated during this study is available from the corresponding author upon reasonable request
